# Overcoming Digital Exclusion during the COVID-19 Pandemic: Impact of Mobile Technology for Survivors of Modern Slavery and Human Trafficking – A Mixed Method Study of Survivors and Support Service Provider Views

**DOI:** 10.1080/23322705.2022.2050991

**Published:** 2022-03-29

**Authors:** Alice Malpass, Kate Garbers, Lauren Saunders, Jeremy Horwood, Hugh McLeod, Eric Anderson, Michelle Farr

**Affiliations:** a The National Institute for Health Research Applied Research Collaboration West (NIHR ARC West) at University Hospitals Bristol and Weston NHS Foundation Trust, UK; b Population Health Sciences, Bristol Medical School, University of Bristol, UK; c Unseen, Bristol, UK; d Rights Lab, Faculty of Social Sciences, University of Nottingham, UK; e BT Group PLC (Former Role)

**Keywords:** Digital inclusion, smartphone technology, survivors of human trafficking and slavery

## Abstract

This collaborative, qualitative study aimed to understand the impact that smartphone technology can have for survivors of human trafficking and slavery in relation to their mental health, well-being and social connections, access to services and levels of independence and isolation. The pilot project was conceived shortly before the COVID-19 pandemic by anti-slavery charity Unseen and the telecommunications company BT, in recognition of the potential of smartphone technology to enhance survivors’ recovery from trauma. BT donated smartphones and SIM cards with 6-month call and data packages that Unseen distributed to survivors they were supporting. Seventy-four survivors received a smartphone; 27 survivors were interviewed and 12 Unseen staff completed a free-text survey exploring perceptions of the intervention. A well-being capability measure (ICECAP-A) was conducted with survivors at the start and end of the project. Researchers analyzed all data, triangulating across data sources. Analysis showed support staff play a key role in the success of the intervention to increase digital inclusion. Smartphones helped survivors develop skills to assist them in their move toward independent living and navigate the systems and services in their environment. The intervention was highly valuable to survivors for support, integration and access to services.

Our findings suggest that suitable technology packages should be assessed for inclusion as standard support for survivors of modern slavery within the UK Government’s National Referral Mechanism (NRM). Achieving this change in NRM policy will go some way to realize the United Nations 2030 Agenda, specifically SDG 3 (Good health and wellbeing for all at all ages), SDG 8 (Decent Work-inclusive and sustained economic growth) and SDG 16 (Peace, justice and strong institutions-inclusive societies and access to justice for all).

## Background

Online access to services such as welfare, health, education, work and more is an essential part of everyday life. However, digital exclusion is an experience for many, particularly those who are socioeconomically disadvantaged. Increasingly, this digital divide means that where services are provided online, or where access points are encouraged to be made online, those with the highest need may be excluded from them (Heponiemi et al., 2020). Digital inequity and digital exclusion have been highlighted as a major contributor to poor mental health outcomes during the COVID-19 pandemic lockdowns in the UK (Watts, 2020). Never before has the general publics’ reliance on technology to maintain a sense of connection or to access and participate in vital social care and health activities been more pronounced. Ability to participate in online digital consultations and access (repeat) prescriptions electronically has become an integral aspect of healthcare provision for much of the population in the UK. Technological exclusion is not a new topic, but for survivors of modern slavery and human trafficking the mental health consequences of digital exclusion during periods of unprecedented social isolation and national lockdowns may be even greater than for the general population. Survivors of modern slavery and human trafficking are one of the most vulnerable groups at risk of complex mental health difficulties, including anxiety, depression, aggression, suicidal ideation and post-traumatic stress disorder (PTSD; Okech et al., 2018). Systematic reviews of the health problems associated with human trafficking highlight that appropriate interventions and support services are urgently needed to address the mental health needs of trafficked individuals (Ottisova et al., 2016). Human trafficking affects all facets of an individual’s life, however there is a lack of research about the complex needs of those who have experienced human trafficking (Munro-Kramer et al., 2020). Research that does explore survivors’ needs illustrates how both essential needs of food, clothing and shelter as well as more complex relational, emotional, psychological and physical needs are present (Dell et al., 2017). Evidence suggests that survivors who perceive less social support indicate more PTSD (Okech et al., 2018). Okech et al. (2018) identify that one critical coping mechanism for survivors is to find social support, which can help reduce stress and psychological or mental health difficulties. To strengthen survivors’ reintegration into the community, it is important to identify and strengthen available social supports and prepare survivors to access these supports and use them to their advantage (Okech et al., 2018).

During the COVID-19 pandemic, the closure of public and community spaces, combined with the provision of health, welfare, education and third-sector support services moving online (as opposed to face-to-face or outreach work), has meant many of the advocacy, legal, social and health services available for survivors have become at best challenging to access and at worst, no longer accessible. Milner (2015) describes three ways in which people are digitally excluded: lack of access because of an inability to pay for devices and their running costs; lack of skills to use digital technology; and lack of motivation to engage in digital technology. Survivors may be digitally excluded in all three ways. Research suggests simply giving people IT equipment or access to it is not enough (Allmann et al., 2021). Allmann (as cited in Watts, 2020) argues that what is needed is human intervention, commitment and care. Any intervention to eradicate digital exclusion for survivors of modern slavery will therefore require an integral support service.

The aim of this study was to assess the usefulness that access to smartphone technology can have for survivors of modern slavery and trafficking from the perspectives of survivors and support staff. We particularly wanted to know the impacts of smartphone technology in relation to survivors’ mental health, well-being and social connections, access to services and levels of independence and isolation.

## Methods

This project was exploratory. It was a pilot study to establish preliminary evidence on whether the provision of smartphone technology, within the context of ongoing third-sector support, can assist trafficking survivors on their pathways to recovery and integration, and support their mental health and well-being.

## Study Design

### The Pilot Intervention

Unseen is a non-government organization that provides specialist support for survivors of slavery and human trafficking, providing a safe place for them to recover from trauma and begin to rebuild their lives. Before the COVID-19 pandemic, Unseen and BT had been in discussion about how technology could be used to benefit and assist recovery journeys of those identified as having experienced exploitation and modern slavery. The study reported here is a collaboration between Unseen, BT and the National Institute for Health Research Applied Research Collaboration West (NIHR ARC West), University of Bristol. BT supplied Unseen with refurbished boxed Samsung Galaxy S7 and Galaxy S7 Edge devices, four packs of smartphone steri-wipes and Pay As You Go SIMs, each with 100 minutes monthly allowance for UK landlines and smartphones, unlimited messages and 4GB of data. Each SIM was credited with £80 ($108 USD) balance and unused data rolled into the next month’s allowance. Unseen distributed smartphone devices to survivors who wanted to take part in the project with training on safe use. It was made clear that this was a pilot project that there was no obligation to use the smartphone as their primary device and that after 6 months there was no commitment to offer further top-ups but that they were able to keep the handset and SIM card. Choosing to participate in the project did not affect access to Unseen support and declining to take part in the research did not impact them in being able to keep the smartphone.

Unseen empower survivors to be able to take responsibility for their own online safety and work with them to identify and develop important life-skills to assist them to use the internet and smartphone devices safely. Online safety training practices are included as part of the general support provided to survivors and this was expanded upon as part of the intervention. An online phone and internet safety session with Unseen covers, for instance: location settings, setting up of new accounts, e-mail, attachments and social media, security and privacy settings, meeting people over the internet safely, managing sharing of personal information, and blocking numbers and contacts, among other issues. Training sessions allowed support workers to work closely with survivors to help them understand the reality of risks and potential consequences of using technology and how to do this safely. Where necessary, these discussions were tailored to the individual to aid risk management planning in line with each person’s own circumstances. This allowed individuals to understand and manage risks for themselves.

### Protective Practices Already in Place

At point of referral Unseen requests that mobile devices are switched off before traveling and arriving at accommodation projects. This reduces the risk of traffickers being able to contact and locate individuals and is viewed as a protective practice, allowing for distance between traffickers and those who have been exploited. Upon arrival at accommodation services, use of personal mobile devices is initially restricted. Survivors are assisted to transfer safe contacts to a temporary phone (provided by Unseen) and a phone and internet safety session takes place within 2 weeks of arrival. Safety sessions are also offered for survivors being supported in the community. Survivors taking part in this study were provided with support throughout the intervention for ongoing safety concerns, as needed. This followed existing Unseen usual safety policy.

## Study Methods

The study used qualitative methods, combining i) one-to-one interviews with survivors, ii) use of the ICECAP-A (ICEpop CAPability measure for Adults) measure of capability wellbeing for adults (Al-Janabi et al., 2012) to assess survivors’ wellbeing at baseline and 6 to 7 months after having received a smartphone, iii) smartphone data usage screenshots and iv) free-text response from an online survey completed by support staff. The study received ethical approval from the University of Bristol Faculty of Health Sciences Research Ethics Committee reference 109984.

### Recruitment and Consent

Following ethical approvals in October 2020, Unseen staff attempted to contact all survivors who had received a BT smartphone from May 2020 who had consented to be contacted to invite them to take part in an interview via video call or smartphone. If survivors read and understood English they were emailed or given a written copy of the information sheet. For those with lower literacy levels or where English was not their first language, information sheets were explained and verbally translated. Before consent was taken, an opportunity to ask questions about the study was given. Unseen staff members read out each statement on the consent form, which was then immediately verbally translated by the interpreter (if one had been requested), one question at a time. Unseen interviewers audio recorded informed consent during the call having gone through each component of the consent form with the participant.

### Data Collection

**One-to-One Interviews with Survivors**. Survivors were interviewed via phone by one of the two Unseen staff who were trained, by the first and last authors, in qualitative approaches to gathering data. Training for staff from Unseen lasted 2 hours and included teaching optimal ways to phrase open-ended interview questions, the difference between mining and mapping interview questions and best practice surrounding the use of a topic guide. The training also covered best practices on gaining informed consent, recording interviews and storing data securely to abide by data protection legislation and ethics protocol. Researchers on the study team were available for ongoing support.

Current risks to the survivors were reviewed in advance of the interview and are on-going as part of general support. Participants’ caseworkers were asked to inform the safeguarding officer of any concerns, which could result in them not being able to participate safely; where concerns were raised, safeguarding measures were implemented. When setting up the interview, interviewers explained the importance of the survivor choosing a time and place when they would feel safe and have privacy to talk freely. Interviewers then checked again, at the beginning of the phone interview, that the survivor felt they were in a safe and private space. If the survivor did not have good English skills, they were asked if they would like a translator to translate the consent form, interview questions and answers. Interpreters did not work for the charity but were organized directly through Unseen’s usual translation services. Through these existing contracts, translators were bound by professional contractual confidentiality agreements. Survivors were familiar with this setup as the same translation service was used when they entered Unseen and accessed their support.

The interviews were audio recorded, with consent, using a digitally encrypted work smartphone that Unseen provided to its staff, or an encrypted digital audio recorder. Interviews were conducted in November–December of 2020. An interview safety protocol was developed to document a clear process for supporting both the interviewee and interviewer. If an interviewee was still in the Unseen service, any issues that arose during an interview could be supported through their key worker. If an interviewee was no longer in the Unseen service, they would be given signposting advice to agencies that may be able to assist. If any safeguarding issues arose, these were forwarded to the Unseen Safeguarding officer as per usual safeguarding processes. Staff conducting interviews could access support from their line manager internally, from the other interviewer or from the university researchers.

Interviewers used a topic guide developed by Unseen staff and university researchers. The topic guide was flexible enough to allow for exploration of issues raised by the participant (see Appendix A).

**Unseen Service Support Staff Survey**. A survey of nine free-text questions was emailed to staff who had insights into survivor experiences (see Appendix B). The survey included information about the project, a consent form and reflective questions. Minimum data on staff roles were requested to provide maximum anonymity for respondents. Two e-mail reminders were sent to all staff.

**Capability Measure**. The ICECAP-A capability measure is a quantitative wellbeing measure for the general adult population designed for use in the economic evaluation of health and social care interventions (Al-Janabi et al., 2012). It defines quality of life in terms of five attributes: Stability: “feeling settled and secure,” Attachment: “love, friendship and support,” Autonomy: “being independent,” Achievement: “achievement and progress,” and Enjoyment: “enjoyment and pleasure.” We aimed to assess the feasibility of using the ICECAP-A measure with survivors in terms of administration and completion in order to understand if it was a potentially useful assessment measure for this population and intervention. Whilst ICECAP-A has been translated into multiple languages, this did not cover all the languages that Unseen service users speak, and some non-English-speaking survivors preferred verbal over written information. Because of this, rather than providing written translations, where needed translators verbally translated the ICECAP-A measure to non-English-speaking survivors.

**Date Usage Records**. Screenshots of data usage were also collected but as this data set was incomplete we have excluded it from the analysis presented in this article. We report full methods, data and limitations elsewhere (Garbers et al., 2021).

### Data Analysis

Qualitative interviews were audio recorded by Unseen without using survivors’ names or other identifying characteristics and university researchers further anonymized and cleaned transcripts of any remaining identifiable data (e.g., if survivors referred to third parties, places). Researchers listened to the qualitative audio files to familiarize themselves with the data. Qualitative interviews were transcribed verbatim. One researcher analyzed all qualitative transcripts and a second researcher double analyzed 15% of transcripts, as per COREQ checklist guidelines for robust qualitative approaches to analysis. For analysis, researchers used a framework created in MS Word to organize the analysis of data under predetermined categories taken from the topic guide, whilst also allowing for new, unanticipated themes to be identified as new (column) headings. Participants were listed in each row, with any relevant data inserted into the corresponding cell under each of the topic guide column headings. This process is akin to the methods described as Rapid Analysis (Vindrola-Padros et al., 2020) but also draws upon first and last author experience of thematic approaches (Braun & Clarke, 2013) and framework approaches (Gale et al., 2013). The online staff survey was a free-text survey, which was also analyzed using rapid analysis techniques, this time using Excel. Analysis included comparing staff survey responses with qualitative verbatim survivor responses in order to identify refutational statements and perspectives.

Quantitative ICECAP-A baseline and follow-up data were analyzed using descriptive statistics, including the mean change in ICECAP-A score. For each attribute, respondents select one of the four levels, where the bottom level represents having no capability and the top level represents full capability. Preference-based numerical tariffs allow differences in overall quality of life to be measured and valued on a scale from 0 to 1, where 0 represents no capability for any attribute and 1 represents full capability for all five attributes (Flynn et al., 2015). In the absence of ICECAP-A data for a suitable comparator group (survivors not provided with a smartphone), the analysis informed feasibility of data collection rather than the impact of the intervention on survivors’ quality of life.

This article has been written up as a descriptive, narrative account, supported by verbatim quotes from interviews and free-text survey responses.

## Findings

From the 74 clients who received a smartphone, 43 were not contactable for the study. Of the remaining 31 survivors approached, 27 agreed to be interviewed. Interviewee age ranged from 24 to 63, with a mean age of 33. Twenty were women, fourteen had experienced sexual exploitation, eight forced labor, two domestic servitude and three undisclosed. The nationality of survivors was broad and included survivors from Pakistan, China, Poland, Nigeria, Albania, Philippines, Romania, Chad, Tanzania, Thailand, Ivory Coast, India, Kurdistan, Britain and Egypt. Nine interviews were conducted with an interpreter. Interview length varied between 8 and 24 minutes. Twelve staff took part in the online staff survey, representing a range of roles and services, including from men’s and women’s safe houses, outreach, resettlement and integration teams. Data extracts are tagged with a unique participant pseudonym, with survivor participants as P1-27 and staff as S1–12.

Qualitative analysis led to the development of the key emergent themes, which we now describe in more detail with verbatim examples focusing upon *Roles of the smartphone for survivors*: (i) Autonomy and personhood; (ii) Parental and work role needs; (iii) Staying in touch with services and support; (iv) Supporting education; (v) Navigating and translating. We then explore survivors experience of *Impacts and Wellbeing* in terms of: (i) Independence; (ii) Connection and isolation; (iii) Interactions with support workers; (iv) Managing stress, anxiety and fear; and (v) Challenges.

## Roles of the Smartphone for Survivors

### Autonomy and Personhood

When survivors were first asked why they had wanted to participate in the Unseen project and receive a smartphone, for some survivors there was a sense of ownership of a [simple] material good that was significant for them: “the phone will be mine” (P1). Whilst most survivors in the study had arrived at Unseen with a basic phone, the device given to them through the project was a smartphone, creating a sense of normality that can be aligned with a healthy sense of personhood:

One client was so very happy to receive a smartphone (one as nice looking and as good as he received). It allows clients to feel more ‘normal’ having a ‘normal’ phone that most people have (rather than a small Nokia that is no longer popular). (S8)

For survivors with a personal history of being forcibly isolated and disconnected from their environment, there is additional weight to the needs expressed around communication and direction. Navigating physical and communicative social worlds as a survivor, supported by smartphone technology, suggests a freedom of movement and expression, which has previously been barred and silenced. One participant said, “I needed a phone because I had just a simple phone, a basic phone, and the new phone helps me translate things because my English is not good and I also find directions on the map” (P6). For others in temporary housing the smartphone provided access to the internet for the first time, making challenging life circumstances just a little bit easier: “No, I don’t have wi-fi because I’m still in temporary house … I have a phone, but there was no internet, so I have this Unseen one because it comes with internet, to help me to make my stuff easy, talking with colleagues, GP, different appointments for myself” (P13).

Staff working with survivors saw access to legal information in their own language as key to being able to empower themselves:

Information regarding the asylum and trafficking legal processes, particularly in other languages are all available online and almost nowhere else. Information about local services can be found online. Without access to this information refugee and asylum seeker and trafficking communities are lost at sea in an unfamiliar bureaucratic structure with no way to empower themselves. (S11)

### Parental and Work Role Needs

For women with children living through a pandemic, navigating the online social worlds was a key need:

Yeah ‘cause um I’m a single mum so I really don’t have much friends in [City] … most of my friends are online, people I’ve never met so it was just like a way to keep me going, to at least … read their stories … and share my stories as well. (P5)

Smartphones provided mothers with a way to access support from both informal and formal sources, including video calls with Project mama and accessing parenting forums. One respondent remarked, “It has really helped share my experience and read other people’s experience on how they are coping with their kids” (P5). And another said, “I had video calls regarding the baby, how to look after the baby” (P6).

Survivors with children needed their smartphones to keep up to date with their children’s education and take part in parent-teacher meetings:

If they want to ask you about your son, if they want you to fill the form, yeah, so they was e-mailing you, and you have to reply to them. The last e-mail, they said they got a meeting with us, but we can’t see each other, so it will be Zoom video. (P13)

For other survivors who were mothers, being able to use their smartphone to occupy their young child had a beneficial impact on the parents’ wellbeing, who was often left alone for long stretches of time with no other source of support:

When it comes to my son as well, it was keeping him busy … As I am with him a lot, you can relax if you give him watching something; you can relax as well, because he will be there, not disturbing you, so it was helpful, for my side. (P13)

Accessing cartoons and educational programming provided both the parent and child with some much needed down time. Access to the internet through the smartphone supported single parents, whose children watched cartoons and movies, “helped me a lot [as a parent]” (P11). However, the data package was often not enough if the smartphone was being used by a single parent, “[when] little one was going in [to phone] it [data allowance] would be finishing quickly … It helps me, but ten pounds doesn’t stay for very long” (P13).

This could lead to a cyclic experience of impoverishment, as parents, already isolated, had to balance their children’s needs to access online entertainment during a pandemic with their own needs as parents to make appointments, and connect online with parenting forums to counteract isolation.

For survivors who were working, it was their workplace environment that prompted their willingness to participate, so they could feel on par with work colleagues. Phones enabled survivors to keep in contact with employers: “Sometimes my work who call me for my job” (P10).

### Staying in Touch with Services and Support

Staff described how the smartphones enabled access to psychological support when services moved online at the beginning of the pandemic:

Many essential services such as counselling and support moved online during the pandemic, VT’s [victims of trafficking] are entitled to counselling as part of their recovery which has only been accessible through a smartphone. Suddenly stopping counselling is considered dangerous for someone’s mental health, having the phones allowed for a continuation of services. (S11)

However, staff also described the lack of access to a big enough data package meant survivors were not always able to get support when they needed it. One explained, “For example, one client regularly ran out of smartphone data and so was unable to contact for support when she needed to” (S12).

In many ways survivors were more easily contactable and able to access online medical services, such as repeat prescriptions. One participant recalled, “Doctor call me sometime and [Name] our support worker she call all the time. And message. Repeat prescription. Making appointments.” (P21).

Smartphones enabled a two-way connection where survivors were able to stay in touch with services, including therapists, counselors, solicitors, lawyers, support workers and healthcare providers; and services were also able to connect with survivors.

### Supporting Education

Survivors involved in education used their smartphones to attend classes and access learning materials: “Yes I go to school and I have to do the course online” (P8). Translation, in relation to studying English, was also facilitated by their smartphone: “Most of all for the English course. I can use the app which translates it from English into Albanian, and this has been helping me a lot … I have YouTube, which I watch a video in relation to my course or other things.” (P11)

For other survivors, they used their smartphones to support self-study and go over material covered during online teaching sessions.

### Navigating and Translating

For survivors learning to move around the city freely for the first time, their smartphones supported them to navigate the city and public transport. A participant explained, “Helped me use Google Maps to find destinations … on Google Maps I did check the bus times and things like that, and it did help me a lot” (P11). For staff working with survivors, being able to independently navigate the city confidently was vital during pandemic conditions, with one saying, “Access to online maps helped increase confidence, especially with limited face to face it’s good for clients to find a new location/appointment independently” (S8).

Translation apps such as Google Translate were used by survivors to facilitate contact with professionals including support workers, solicitors and healthcare providers: “I use Google Translate for letters received from Dr and social worker” (P6). In reaching out to other mothers on online platforms, it was important to one survivor to not look foolish, so she used her smartphone translate app to be proficient:

I was trying to write something online … I was trying to make a comment on one of the mother’s Facebook group and we were talking about choking and then I got confused on whether there is C before K or no C and I had to ask Google how to spell choking [laughs] … *Just to be sure I don’t make a fool of myself [laughs]*. (P5)

Anxieties about mis-spelling were eased through access to technology, facilitating a sense of belonging to a group. Staff also described the importance of being able to continue access to learning English and framed this in terms of integration, empowerment and safety:

Clients access ESOL language classes which are extremely important for a clients integration into the UK, their empowerment and feeling of safety in the UK, these services have been able to continue online which clients have accessed. (S11)

Though survivors did not describe using Google Translate with other survivors they were living with, staff described the impact of Google Translate on day-to-day peer support:

The residents are able to communicate with each other better. We have service users from different countries staying with us and whilst staff facilitate interpreter calls for support work they are unable to do this for day-to-day peer support. (S3)

**Figure 1.**
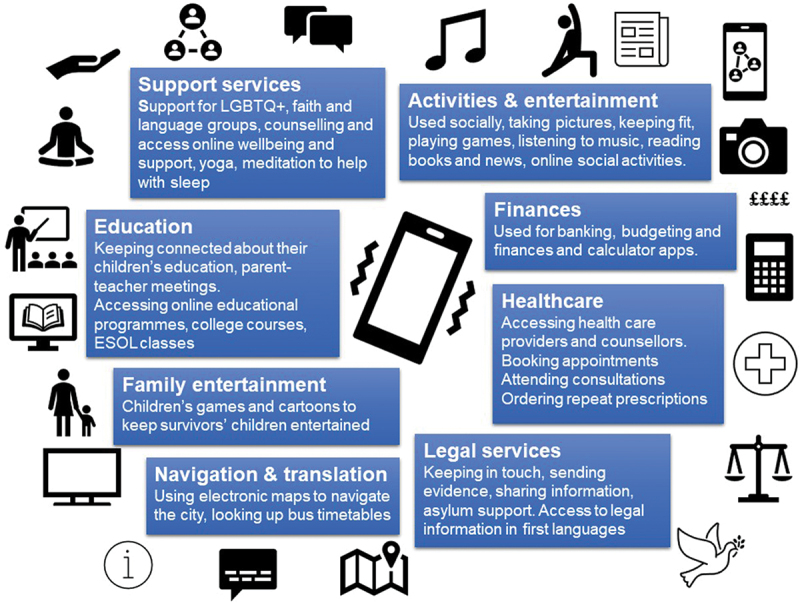
How smartphones were used.

Figure 1 summarizes the different uses of smartphones that survivors highlighted.

## Impacts and Wellbeing

### Independence

Survivors were asked whether their smartphones had made any difference to their independence and were able to describe an increased ability to do things for themselves (as described above). A few survivors did not immediately understand the word “independence” and so did not answer this question directly, despite describing throughout their interviews various ways their smartphones enabled more ability to do things for themselves. Interviewers iteratively developed the topic guide and in later interviews, altered the wording to make the meaning more understandable (asking if the phone enabled people to be able to do more things for themselves – see Appendix 1). In contrast, the staff working with survivors often used the concepts of independence, emphasizing self-determination (able to book their own appointments) with less need to contact support workers. One staff member explained, “Yes – service users have been able to access emails for themselves and look up directions on their phones, before the Unseen project they often came to the office to request staff support with this” (S3).

Improved ability to contact support workers and send information via e-mail or WhatsApp made life easier both for survivors and the service support staff:

Access to picture sharing makes supporting these communities easier, they can send a photo of a letter to their support worker rather than having to pay bus fare to travel across the city to meet someone in person for support with reading the same letter. This saves time and money for clients and our organisation. (S11)

As well as the added benefits of video calls, staff recognized the added autonomy now available to the survivors they were supporting. One staff indicated, “I think it has helped. My client is able to text/call if she needs to change our appointment or if she is concerned about something, she can call straight away rather than having to wait until I contact her” (S4).

### Connection and Isolation

When asked whether their smartphone had made it easier to be in touch with family, most survivors said they had no family. Survivors who did have connections to family were pleased with the increased access they now had: “Sometimes I ring my country, I spoke with my family in the camera.” (P10). When asked if their smartphone had helped with feelings of isolation or loneliness, survivors did not tend to report major shifts in mood, instead smartphones seemed to provide a welcome distraction:

Going on Facebook has really helped me connect because without internet I would be like ‘oh I’m bored’, at home and not having much to do for myself so I read some stories, I read some posts which really makes things easier for me. (P5)

The technology made day-to-day life more bearable, even connecting at times, but it did not always fundamentally help overcome feelings of loneliness:

I put my video and then I just spend time watching … I don’t really get text messages because I really don’t have friends. Even these people they call me, they’re just people like my caseworker. Just I don’t have many people to talk to. (P26)

In contrast to the data from survivors, staff working in outreach and safe house settings believed access to smartphone technology, “reduces isolation massively … helping with mental health [by] distract[ing] from overthinking” (S2). Staff perceived that isolation had been alleviated by increased contact with community church groups in Zoom, with friends and family and with other survivors who are from different cultures and languages. One participant explained, “They have been able to contact friends they have made while being in the service to support each other/talk online/arrange to meet up, helping reduce isolation” (S4).

Staff also viewed the ability to communicate with others as a lifeline, helping overcome isolation and vulnerability:

Clients without smartphones have spent lockdown in houses without internet, shared with people who do not speak the same language. They have been extremely vulnerable and isolated during the pandemic. [Having] a phone and access to the internet is a great improvement to their wellbeing and access to services. (S11)

### Interactions with Support Workers

Whilst survivors only reported positive impacts of having a smartphone on their interactions with Unseen staff, staff provided a more mixed response, with two staff members reporting the smartphones had not changed interactions with survivors. Most staff reported positively, describing improvements in carrying out their role as support workers, building trust and rapport:

Clients having access to smartphones has made working and particularly working during the pandemic much easier. File sharing through email and picture messages saves us vast amounts of travel time, room hire and COVID risk. Having access to video calls has enabled us to ‘meet’ new clients more fully when f2f [face-to-face] meetings have been impossible which has benefited building of trust and understanding within our support role as well as important for identification in an emergency. For existing clients, it has allowed a level of emotional support not possible just over the phone. (S11)

Both survivors and support staff described only positive impacts on communication with other professionals and service providers:

Again, much improved, they are now able to give out their own phone numbers to other professionals involved in their care, before all [outreach] calls came through the [safehouse] office and messages passed on through a support worker, the project has ‘cut out the middleman’ a little and led service users to speak directly with professionals on their own timeframes and not when staff are around to facilitate this. My GP app also used lots, this used to be done by staff via computer in office. (S3)

### Managing stress, anxiety or fear and safety

Only four survivors explicitly mentioned using their smartphones to help them manage stress and anxiety. For one survivor, their smartphone was compared to a best friend, reducing their sense of stress by putting them in touch with others during periods of increased restrictions in the pandemic. For others, their smartphones supported stress management by providing access to online supportive classes and self-help apps:

Because I got stress for every single minute, but if I have my phone sometimes … take yoga or … to feel happy … something funny [to watch] … if I using like that, after I feel better, you know. If I don’t have phone I feel crazy, honestly. (P12)

Another commented, “I use this website Calm about meditating because I struggle with sleep” (P18). One survivor discussed living in fear of the person who had exploited her, fearful of them locating her when released from prison. For this woman, her smartphone represented a source of safety, because others whom she trusted could check on her. She said, “Well the most useful thing … my auntie, when she goes to work, she still calls me to check on me, how I am, because she knows I feel scared” (P23). She spoke at length about her experience of hyper vigilance and the important practical and symbolic role the smartphone played in her sense of safety:

Because when I go outside in the street, I mean I always have to look around me, keep an eye on everything, right, left, behind me because I know when that man comes out of prison, 100% is gonna come searching for me, so I always have that presence all the time in my mind, so that’s why the phone is very useful, most of all is call the police in case that happens. (P23)

The smartphone provided gave her a valuable way to feel safer. It meant instead of locking herself up inside, she felt brave enough to venture outside. She also used her smartphone to make contact when she had ventured outside but had started to feel panicky:

Yes, because to be able to call a relative when you are feeling frightened or you are feeling scared or call a friend, saying oh I am feeling scared right now, so then they are able to check and make you feel calmer, so that is a great help. (P23)

Two staff members reported that for survivors with substance misuse issues, different safety issues emerged:

Due to the vulnerability of our service users, we have also seen the odd incident of security issues with the phones. Staff have provided safety sessions on the phone and personal safety but some (a small number) clients with complex needs such as drug and alcohol dependency have got themselves in circumstances where their phone has been stolen or damaged. (S3)

One staff member suspected that there may have been occasions where survivors had sold or exchanged their smartphones for financial benefit. We also asked survivors about any potential safety risks posed by having the smartphones, the two issues that survivors highlighted were related to technical advice and data security. One said, “When it comes to downloading stuff, I ask someone to help me. I’m no good in downloading stuff” (P13). The other similarly commented:

I didn’t know how much the company have got access, so that was much more about the pictures, so I didn’t know if there is anything like a link or iCloud, but the company that gave out the phones … like I didn’t know how much they have got access on the phone? (P14)

These issues raised highlight the need for ongoing support and advice in relation to being able to manage access to digital technology.

### Challenges

The major challenge reported by both survivors and staff was data running out, leading to increased anxiety for survivors, “at times creating barriers between staff and clients” (S9). When data ran out, emotions and confusion over why this was were placed on those facilitating both the project and their support. This added to the emotional labor of service support staff:

Data running out has been a challenge for a number of service users e.g. if a service user is in the middle of a task/online support session and their data runs out unexpectedly this can lead to increased anxiety and stress. Even those who are using their phones for recreational use such as watching music videos and movies have found this difficult. Those with complex needs have found this aspect particularly difficult to understand and have often turned to staff with frustrations and confusion that the support worker has needed to assist them with. (S3)

The amount of data supplied with the smartphones was agreed prior to the pandemic beginning and was thought to be sufficient. One staff member viewed the data package as unrealistic for the survivor’s circumstances, many of whom don’t have wi-fi, have family or friends overseas and during lockdown only had access to their smartphone for entertainment or activity. Whilst survivors talked about not having enough data or it being used up very quickly, none of the qualitative interviews contained descriptions like those given by staff. Further analysis of data usage statistics is available in Garbers et al. *(2021)*.

## Capability Measure Analysis

**Figure 2.**
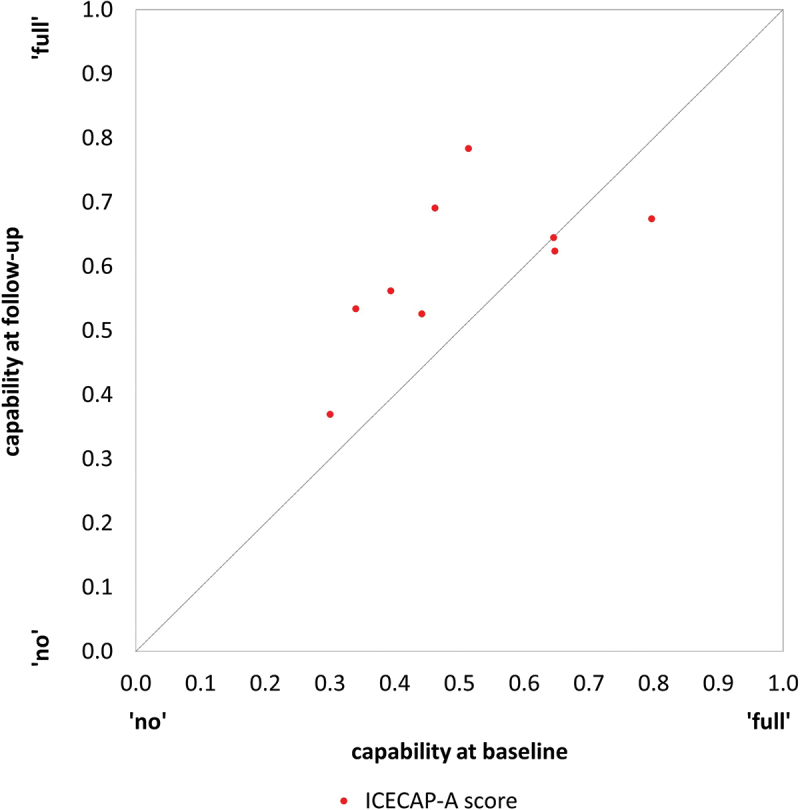
ICECAP-A scores at baseline and follow-up (n = 9).

Baseline ICECAP-A data for 78% (21/27) of participants were available for analysis. A response was recorded for all but one of the attributes of the 21 ICECAP-A forms (i.e., 99%, 104/105). On the scale of 0 to 1, where 0 represents no capability for any attribute and 1 represents full capability for all attributes, the mean ICECAP-A score for the 20 completed forms was 0.561 (range 0.300 to 0.849, median 0.577). Follow-up ICECAP-A data were available for nine survivors, 33% (9/27) of those interviewed, and the length of time between baseline and follow-up data collection was between 6 and 7 months. For these survivors, the mean score increased by 0.097 from the baseline of 0.512 to 0.601 at follow-up. The baseline and follow-up scores for these survivors are plotted in Figure 2. Six survivors experienced an increase in ICECAP-A score, two experienced a decrease and one experienced no change (see Figure 2). These survivors span a wide range of capability experienced.

**Figure 3.**
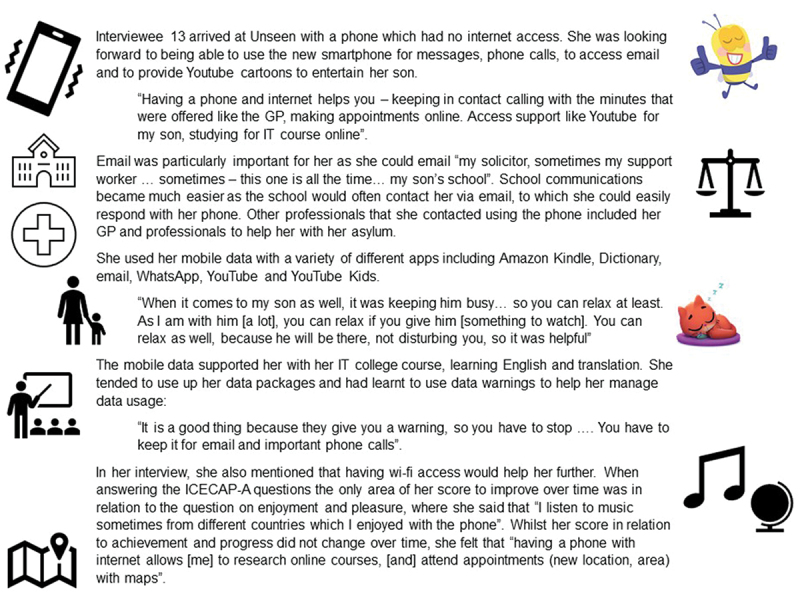
Case study of participant 13.

Five survivors provided additional comments on how the ICECAP-A attributes felt relevant to their situation and how phones made a difference to their capability. People who provided comments on the ICECAP-A questions did not need a translator for the interview, which may reflect their greater understanding of the English language. Survivors gave examples that illustrated how having a phone related to their experiences of “feeling settled and secure” and were able to relate their experience of the “love, friendship and support” attribute to having a phone. Some survivors highlighted aspects of their life relating to the “independence” using the example of Google Translate to improve understanding which increased their independence and “enjoyment and pleasure” attributes, which their phone had directly enhanced their capability to experience. One participant commented, “It’s been so helpful to access games and videos for my children, thank you so much, it really did come at the perfect time” (P14). Further quotations about how survivors interpreted and understood the ICECAP-A measure in relation to the impact of having a smartphone are reported in Garbers et al. (2021). Figure 3 provides an example of a case study that brings together the different data sources, to illustrate one survivor’s use and the impacts of the smartphone.

## Discussion

Our findings suggest that suitable technology packages should be assessed for inclusion as standard support for survivors of modern slavery within the UK Government’s National Referral Mechanism (NRM). Including technology packages within NRM policy will begin to realize the United Nations 2030 Agenda. For example, in terms of the impact of smartphones they could support mental health, well-being and social connections. Receiving a smartphone had socio-symbolic value, providing a sense of normality and strengthening a sense of personhood through experiences of belonging and an increased sense of autonomy. These findings specifically fulfill the UN 2030 Agenda item-SDG 3 (Good health and wellbeing for all at all ages).

In terms of usefulness, smartphones were used to access social platforms, support study, contact friends and family, contact legal, health and support services, be contactable to employers and their children’s school. Often utility and impacts upon wellbeing overlapped, for example, smartphones supported independence and help seeking behaviors with online maps and translation apps making contact with professional services easier as well as facilitating collection of food parcels. These findings suggest the multiple ways in which including technology packages within NRM promises to fulfill the UN 2030 Agenda item, SDG 16 (Peace, justice and strong institutions-inclusive societies and access to justice for all).

In terms of the impact of a smartphone upon mental health, well-being and social connections for survivors who were parents, smartphones supported parents by connecting them to other parents, child healthcare providers and schools whilst also providing some much-needed children’s entertainment (and parental respite). However, the data package in the project was designed prior to the COVID-19 pandemic and assumed adequate for “usual use.” Consequently, parents in this study were left to choose between using data allowance for their children’s needs or their own.

The comparison of survivor data and staff data raised some interesting contrasts when exploring the topic of reduced isolation. Survivors did not tend to report major shifts in feelings of loneliness, describing instead technology making life more bearable, providing a welcome distraction from boredom rather than a solution or remedy to isolation. In contrast, staff viewed access to technology as a lifeline, helping overcome isolation and improving mental health by distracting from overthinking. One possible explanation for this mismatch in the two perspectives is that staff saw the impact of having no access to smartphone technology on survivors in the period leading up to the study and made comparisons between those digitally excluded and those with increased digital inclusion via the pilot project. There was also disagreement in the data between staff on the impact of smartphone technology on client-staff communication. Most staff found file sharing through e-mail and picture messages saved them vast amounts of travel time, room hire and COVID-19 risk, so viewed the pilot positively. Safehouse staff saw more first-hand the displays of emotion when data ran out, as they were often on site when this occurred and witnessed first-hand the impact and challenge of the small data package. This initially increased staff emotional labor when responding to a client’s heightened anxiety when suddenly without data. Survivors were able to share frustrations if data ran out, and staff supported survivors with emotional support to reassure, manage emotions and offer practical support on what to do in future to manage the data allowance or how best to deal with this situation should it happen again. Survivors also learnt how to manage their data allowance better as time went by.

The impact of the smartphones on issues of safety was more complex. For one survivor, having a smartphone definitely increased her sense of safety, whilst some staff were more aware of safety risks caused by the small data package, leading to sudden inability to continue counseling or the inability to contact services when most in need.

### Re-considering the Evidence from this Study

Professionals and campaigners are both keen to champion the role of digital technology in the UK, for example, Graham Kendall, Director of the Digital Healthcare Council argues that “the spotlight on digital health may lead to profound long-term changes for our health services” (as cited in Watts, 2020, p. e396). Fulfilling this vision for the most vulnerable in our society, such as survivors of modern slavery and human trafficking, means gathering evidence on how digital inclusion projects, such as the one described here, overcome the different types of digital exclusion. To what extent does study evidence demonstrate the intervention has overcome (i) lack of access because of an inability to pay for devices and their running costs; (ii) lack of skills to use digital technology; and (iii) lack of motivation to engage in digital technology?

### Lack of Access

Our findings suggest that suitable technology packages should be assessed for inclusion as standard support for survivors of modern slavery within the UK Government’s National Referral Mechanism (NRM). Whilst the project has provided good evidence that a collaboration between a third-sector organization and the IT industry is a feasible pilot, for a long-term sustainable approach, this intervention needs to be integrated into funded support packages providing the essential aspects of care, human connection and commitment, which Allmann et al. (2021) highlights as paramount. The pilot project, in its current format, did not overcome the barriers to running costs associated with data packages. Further recommendations are discussed below.

### Lack of Skills

All survivors in this study had prior experience with mobile technology but not smartphone technology. The pilot project has provided good evidence that the key to success in overcoming digital exclusion, is not the provision of equipment alone but the ongoing human relationships with support workers who can assist the setting up of smartphone devices for those who do not yet speak English or who are new to smartphone technology, supporting and navigating survivors through any technical or safety concerns. Whilst it is clear that the data packages were not adequate for the needs of this digitally excluded group, this may have been compounded with a lack of understanding or skills in how to manage the data allowance, combined with increased usage during the COVID-19 pandemic.

### Lack of Motivation

There was no evidence in the pilot data that survivors lacked motivation to engage with digital technology. However, no interviews were secured with survivors who had exchanged or sold their device, nor with Unseen survivors who refused to be interviewed or were uncontactable. There could be several reasons for why this was: Unseen survivors may have experienced a lack of motivation to engage with digital technology, experienced a change in circumstances or did not want to be involved with the research element of the project. As a result, we have not been able to capture everyone’s experiences of receiving a smartphone nor do we have access to narratives of dis-engagement.

## Recommendations

### Including Access to Technology as Part of Standard Survivor Support Packages

Many advocacy, health and legal services are likely to continue to have their primary services online after the COVID-19 pandemic. Survivors of modern slavery and trafficking should not be expected to live without this support. The pilot project has provided substantial qualitative evidence to recommend the provision of suitable technology packages (and potentially the rolling out of this project nationally), to ensure digital inclusion is part of survivor rehabilitation packages, with the proviso that third-sector service support staff play a key role in the implementation of digital inclusion packages, including training in digital safety and device set up.

### Access to Unlimited Data for Survivors

Increased data capacity, specifically unlimited data packages, would improve the impact of any intervention. For greater consistency in accessing educational, health and legal services, a larger data package is needed than that used in the pilot project. Unlimited data would enable survivors to access everything they needed to rather than having to make choices about what they access. This would avoid exacerbating survivor stress, frustration and anxiety. An unlimited data package would also mean survivors do not have to use any of their weekly subsistence money on smartphone needs and can instead use this on food and travel. A key criterion for a national rollout (in which access to a smartphone is standardized as part of NRM support packages) is the provision of access to unlimited data.

### Partnership Working and Evidence Base

Government sponsored networking events can facilitate organizations to make connections and work together to develop effective solutions to ensure equitable and appropriate provision of devices and access to technology. Following the exemplary example reported here, the government, the NGO sector and business should work together to navigate how recommendations described above (access to technology and provision of unlimited data) can be practically implemented, funded and made sustainable. Collaborations should always include representatives of the survivor community wherever possible.

## Strengths and Limitations

This is the first study to report on a digital inclusion intervention with this population during the COVID-19 pandemic. It is also one of the few studies to combine and contrast survivor and staff views and experiences. The study found that it is feasible to collect data on well-being from survivors, although further steps would be warranted in further research to improve data completeness. There are considerable ethical challenges for researchers seeking to understand survivors' perspectives, ensuring that any involvement in research does not re-traumatize individuals (Lockyer et al., 2020). Unseen staff were viewed by the project team as being best placed to conduct interviews, based on the fact they already knew the situations survivors were facing and had experienced. However, there is potentially a risk of bias resulting from Unseen staff occupying “dual roles” – which in this case refers to one interviewer from Unseen collecting interview data being known to the interviewee in a *previous* capacity, whilst also being the “non-professional researcher” exploring the interviewee’s experience of the BT smartphone intervention. At the time of the interview, no survivors were receiving support from the staff member conducting the interview. Training provided by the first and last author explored optimal ways to mitigate bias resulting from “dual roles.” Future research faced with this same ethical dilemma should refer to the WHO guidance on ethical and safety recommendations (Zimmerman & Watts, 2003).

A second limitation of this study is the short duration of interviews with survivors. However, compared to other studies with survivors of human trafficking, the strength of the study is that our sampling strategy was wide and inclusive, with Unseen staff attempting to contact all Unseen survivors who had received a smartphone and who had consented to be contacted about the study. This meant the current study was able to recruit 27 participants, a much larger sample than other studies with this population (Lockyer et al., 2020; Mumey et al., 2021). A fuller discussion of some of these limitations and strengths, including further reflections on working collaboratively with a third-sector organization, an industry leader in smartphone technology and experienced university researchers is reported elsewhere (Garbers et al., 2021).

A third limitation was that due to budget constraints we were not able to check for consistency in translations and interpretations of the interview questions or ICECAP-A measure.

## Future Research

More research is needed to further understand survivor needs in relation to technology. During this project, we became aware of other organizations working on a similar topic and there have been calls for further research in this area (Modern Slavery Policy and Evidence Centre, 2021). Our research team is exploring ways to work collaboratively to have maximum impact from this research. Alongside our work reported here, there have been several pieces of research published recently about survivors’ wider support needs, for example, Preparing for Impact (Semione, 2020), Going Places (Davy, 2020) and Closed Doors (Hibiscus Initiatives, 2020). A systematic review of this evidence base is now needed to synthesize the impact of different survivor support packages on survivor wellbeing and health. Findings can then be shared with the sector to assist policymakers as they work on NRM transformation programs and support policies for survivors.

## Conclusion

Digital inclusion supports independence and integration in the community for survivors of modern slavery and human trafficking and their children, with support staff playing a key role in the success of any intervention to increase digital inclusion. Access to a smartphone and data package helped survivors develop skills to assist them in their move toward independent living and an understanding of the systems and services in their environment. Support staff experienced both benefits from those they were supporting being *digitally included* (streamlining support tasks and increased safety during a pandemic) as well as disadvantages (such as intermittent increases in tension in staff–survivor relationships and therefore increased emotional labor). The COVID-19 pandemic was not the catalyst for this project, although it sped up its implementation. The benefits of having access to a smartphone and data, although amplified during a pandemic, arguably are present in *normal* times. Access to technology by itself should not be seen as a standalone solution but should be considered a feasible and necessary element of the support packages offered to survivors, so they can move forward in their journeys of recovery and reintegration.

## Data Availability

Due to the sensitive nature of this interviewee group, anonymized study data are not available to preserve confidentiality.

## Partners and Responsibilities

**BT** – provision of all smartphone devices, data bundles, SIM cards and funding to cover project administrative costs.

**Unseen** – management of project, data capture, writing of interim report and co-authoring of evaluation report.

**NIHR ARC West, Bristol Medical School, University of Bristol (UoB)** – Research funding, ethics and governance, research methods advice, analysis of data, staff training and co-authoring of evaluation report and academic publications.

## Appendix A: Interview Topic Guide

1. We would like to get some background information on who we’re speaking with, if you’re happy to share these details.
  - Would you mind sharing with us your age, gender?
  - How would you describe your ethnic background?
  - How long have you been using Unseen services?
2. Why did you want to have a phone? What did you find most difficult about not having a phone?
3. Have you had any previous experiences of using mobile technology?
4. What have been the most helpful things that you have used the phone for?
5. How has the phone made a difference to you?
  - Prompts: do you feel able to do more things for yourself? Has it made a difference to your feelings of independence? (in later interviews this changed to “Do you feel you can do more things for yourself?”)
6. Have you been able to talk to friends and family more now? Prompts:
  - With Family (in UK/abroad) – whats app, face time, phone call, SMS, call, other
  - With friends (in UK/abroad) – whats app, face time, phone call, SMS, call, other
7. What’s that been like? (Prompts: “tell me more”, “can you say more about that”)
8. Who, if anybody, have you been able to connect with anyone, that you would not have, without a phone?
9. Have you felt more or less supported now you have a phone? Why?
10. Do you feel more or less isolated with your phone? Why?
11. Have you had any problems in using the phone? Prompt: did you need more guidance to help you use it? Are there things you don’t understand about the phone?
12. What, if anything, has worried you about using the phone?
13. What, if any, concerns about your own safety when using the phone?
14. What services have you accessed using your phone? Prompt: If yes to any of the below, what difference did that make to you? Did it help you? How?
  - Support worker – how? (e.g., whats app, face time, phone call, SMS, call, other)
  - Counselor/Counseling sessions – how? (e.g., what’s app, face time, phone call, SMS, call, other)
  - Health services, e.g., GP, medical (other)
  - Have you made any other appointments using your phone? E.g., College, Job Seeking, Legal, dentist, optician, banking
15. Have you used the phone for?
  - Google Translate (Prompt: If yes, what difference did that make to you? How did it help?)
  - Information (Prompt: If yes, what difference did that make to you? How did it help?)
    - Looking up opening times – shops, college, work, leisure activities
    - Journey timetables – bus, train
    - Accessing education
    - If yes, what difference did that make to you? Did it help you? How?
  - Entertainment
    - Social media – twitter, FB, TikTok, e-mails, other. Has that helped you to connect with people? How?
    - YouTube, Netflix, Other

## Appendix B: Survey Questions for Support Staff

1. What is your job title and which primary project do you work in?
2. Have you seen any benefits in the smartphone pilot for service users? If so, please give examples.
3. Have phones helped service users to be more effectively supported during lockdown and the pandemic? If so how? 4 Have you seen any challenges in the smartphone pilot for service users? If so, please give examples?
4. In your opinion, how has having access to a phone and data changed client interaction with: a Yourselves b Other professionals involved in their support c Their personal relationships & contacts?
5. Have you observed clients doing anything differently because of their phones, e.g., using electronic maps, translation services, looking up bus times, communicating with friends/ family, other?
6. Have you seen any other emotional or practical impacts that the phones have had on clients, not covered by the above questions?
7. Have you identified any safety concerns specified to service users’ access to a phone?
8. Would you suggest doing anything differently if phones were given out to clients again?
9. Is there anything else we should consider as we review this project and make policy recommendations?

## References

[cit0001] Al-Janabi, H., Flynn, T. N., & Coast, J. (2012). Development of a self-report measure of capability wellbeing for adults: The ICECAP-A. *Quality of Life Research*, 21(1), 167–176. 10.1007/s11136-011-9927-2 21598064 PMC3254872

[cit0002] Allmann, K., Blank, G., & Wong, A. (2021). Libraries on the Front Lines of the Digital Divide: The Oxfordshire Digital Inclusion Project Report. Centre for Socio-Legal Studies. University of Oxford. https://www.law.ox.ac.uk/research-and-subject-groups/oxfordshire-digital-inclusion-project

[cit0003] Braun, V., & Clarke, V. (2013). *Successful qualitative research: A practical guide for beginners*. Sage.

[cit0004] Davy, D. (2020). Going places: Journeys to recovery - A study on the benefits of providing survivors in the UK National Referral Mechanism with funded transport. University of Nottingham, Rights Lab. https://www.nottingham.ac.uk/research/beacons-of-excellence/rights-lab/resources/reports-and-briefings/2020/december/going-places-journeys-to-recovery.pdf

[cit0005] Dell, N. A., Maynard, B. R., Born, K. R., Wagner, E., Atkins, B., & House, W. (2017). Helping Survivors of Human Trafficking: A Systematic Review of Exit and Postexit Interventions. *Trauma, Violence, & Abuse*, 20(2), 183–196. 10.1177/1524838017692553 29333961

[cit0006] Flynn, T. N., Huynh, E., Peters, T. J., Al-Janabi, H., Clemens, S., Moody, A., & Coast, J. (2015). Scoring the Icecap-a Capability Instrument. Estimation of a UK General Population Tariff. *Health Economics*, 24(3), 258–269. 10.1002/hec.3014 24254584 PMC4322472

[cit0007] Gale, N. K., Heath, G., Cameron, E., Rashid, S., & Redwood, S. (2013). Using the framework method for the analysis of qualitative data in multi-disciplinary health research. *BMC Medical Research Methodology*, 13(1), 1–8. 10.1186/1471-2288-13-117 24047204 PMC3848812

[cit0008] Garbers, K., Malpass, A., Horwood, J., McLeod, H., Anderson, E., & Farr, M. (2021). Impact of smartphone technology for survivors of modern slavery and human trafficking during COVID-19 pandemic: A mixed method study. Unseen and University of Bristol. https://www.unseenuk.org/wp-content/uploads/2021/10/FINAL-Unseen-BT-Evaluation-report_Technology-report_17MAY.pdf 10.1080/23322705.2022.2050991 PMC11256988 39036779

[cit0009] Heponiemi, T., Jormanainen, V., Leemann, L., Manderbacka, K., Aalto, A.-M., & Hyppönen, H. (2020). Digital Divide in Perceived Benefits of Online Health Care and Social Welfare Services: National Cross-Sectional Survey Study. *Journal of Medical Internet Research*, 22(7), e17616. 10.2196/17616 32673218 PMC7381057

[cit0010] Hibiscus Initiatives. (2020). CLOSED DOORS Summary Report Inequalities and injustices in appropriate and secure housing provision for female victims of trafficking who are seeking asylum. https://hibiscusinitiatives.org.uk/wp-content/uploads/2020/12/2020_11_24-HI_Closed-Doors_Summary-Report_FINAL_DIGITAL.pdf

[cit0011] Lockyer, S., & Koenig, C. J. (2020). At the Intersection of Method and empowerment: Reflections from a Pilot Photovoice Study with Survivors of Human Trafficking. *Journal of Human Trafficking*, 6(1), 1–20. 10.1080/23322705.2020.180930032190715

[cit0012] Milner, H. (2015). Local + Digital + Scale: A Mass Movement for Digital Inclusion. In K. J. Andreasson (Ed.), *Digital Divides: The New Challenges and Opportunities of E-Inclusion* (pp. 217–246). CRC Press.

[cit0013] Modern Slavery Policy and Evidence Centre (2021). Call for research: Distributed technology for survivors. https://modernslaverypec.org/latest/call-research-distributed-technology

[cit0014] Mumey, A., Sardana, S., Richardson-Vejlgaard, R., & Akinsulure-Smith, A. M. (2021). Mental health needs of sex trafficking survivors in New York City: Reflections on exploitation, coping, and recovery. *Psychological Trauma*, 13(2), 185–192. 10.1037/tra0000603 33119348

[cit0015] Munro-Kramer, M., Beck, D., Choi, K., Singer, R., Gebhard, A., & Carr, B. (2020). Human Trafficking Victim’s Service Needs and Outcomes: An Analysis of Clinical Law Data. *Journal of Human Trafficking*, 6(1), 95–108. 10.1080/23322705.2019.1574476 37600928 PMC10438864

[cit0016] Okech, D., Hansen, N., Howard, W., Anarfi, J. K., & Burns, A. C. (2018). Social Support, Dysfunctional Coping, and Community Reintegration as Predictors of PTSD Among Human Trafficking Survivors. *Behavioral Medicine*, 44(3), 209–218. 10.1080/08964289.2018.1432553 30020868

[cit0017] Ottisova, L., Hemmings, S., Howard, L. M., Zimmerman, C., & Oram, S. (2016). Prevalence and risk of violence and the mental, physical and sexual health problems associated with human trafficking: An updated systematic review. *Epidemiology and Psychiatric Sciences*, 25(4), 317–341. 10.1017/S2045796016000135 27066701 PMC7137602

[cit0018] Semione, J. (2020). *PREPARING FOR IMPACT How we can overcome barriers and cultivate a culture of collaboration, understanding, and respect to achieve impact on survivor support*. Independent Anti. Independent AntiSlavery Commissioner https://www.antislaverycommissioner.co.uk/media/1433/iasc-review-preparing-for-impact-july-2020.pdf

[cit0019] Vindrola-Padros, C., Chisnall, G., Cooper, S., Dowrick, A., Djellouli, N., Symmons, S. M., Singleton, G., Vanderslott, S., Vera, N., & Johnson, G. A. (2020). Carrying out rapid qualitative research during a pandemic: Emerging lessons from COVID-19. *Qualitative Health Research*, 30(14), 2192–2204. 10.1177/1049732320951526 32865149 PMC7649912

[cit0020] Watts, G. (2020). COVID-19 and the digital divide in the UK. *The Lancet Digital Health*, 2(8), e395–e396. 10.1016/S2589-7500(20)30169-2 32835198 PMC7384786

[cit0021] Zimmerman, C., & Watts, C. (2003). *WHO ethical and safety recommendations for interviewing trafficked women*. World Health Organization. https://apps.who.int/iris/bitstream/handle/10665/42765/9241546255.pdf

